# An Overview of 3D Bioprinting as a Novel Strategy in the Field of Tissue Engineering

**Published:** 2018

**Authors:** Somaieh Kazemnejad

**Affiliations:** Reproductive Biotechnology Research Center, Avicenna Research Institute, ACECR, Tehran, Iran

Tissue engineering and regenerative medicine have typically matured from benchtop ideas to commercially applicable products in the clinic 1. However, despite of typical advances in tissue engineering field, some limitations such as no reproducibility, no control of structure geometry including pore size and pore distribution and no integrity of cell distribution and migration in the construct have impelled the scientists into bioprinting technology. The most advantage of 3D bioprinting sounds to be precise fabrication of 3D deposition with controlled geometric structure and cells distribution 2. Over the past decade, lots of researches in bioprinting of different tissues and organs has been carried out using different bioprinting modalities particularly inkjet based printing for skin tissue engineering and extrusion based printing for 3D depositions like bone, cartilage, heart, liver and heart valve. The key factor in extrusion-based bioprinting is bioink preparation, cell encapsulation in the bioink and bioprinting procedure. Indeed, preparation of bioink with appropriate gelation rate, suitable mechanical strength and elasticity which preserve cell viability and proliferation is the most challenge of bioprinting technology. So far, different strategies such as dual bioink cross-linkers, multi-step polymerization and using of core-shell nozzle have been reported to improve viability, quality and functionality of the printed product 3. However, some issues including creation of constructs supporting *in vivo* vascularization, scaling up tissue constructs and in situ bioprinting have been remained to resolve. A few bioprinting products have been commercialized especially in orthopedic and skin tissue engineering fields and given the fast development of this industry over the past years; it supposed that the bioprinting products will eventually take a big proportion of the medical market to help patients suffering from a wide range of diseases in the future.
